# Characterization of an extensively hydrolyzed whey infant formula with a low bitterness

**DOI:** 10.1186/2045-7022-3-S3-P132

**Published:** 2013-07-25

**Authors:** M Rapp, C Martin-Paschoud, S Nutten, C Blanchard, A Panchaud, A Jarvi, M Kuslys

**Affiliations:** 1 Nestlé PTC Konolfingen, Konolfingen, Switzerland; 2 Nestlé Research Center, Lausanne, Switzerland; 3 Nestlé Health Science S.A, Lutry, Switzerland

## Background

Numerous extensively hydrolyzed cow’s milk protein based infant formulas are commercially available for the dietary management of symptomatic cow’s milk protein allergic infants. The production process of extensively hydrolyzed formulas has been improved in the last decade to meet even stricter characteristics in term of peptide size and allergenicity. However, the extent of the hydrolysis process is usually associated with a detrimental impact on palatability. Acceptance of product by mothers and infants is key for compliance and thus efficacy. We aimed at comparing the peptide size distribution and allergenicity of several leading commercial whey based and casein based hydrolyzed infant formulas and at defining how the level of hydrolysis and recipe impacts sensory profile.

## Methods

Size exclusion chromatography was used to characterize the peptide size distribution, beta-lactoglobulin (βLG) was quantified by ELISA and rat basophil cell leukemia cell line (RBL) assay was used to assess cross linking activity of the residual βLG. The sensory profile (comparative profile, one sample versus a reference) was carried out with a trained professional panel of 11 adult subjects.

## Results

The whey based extensively hydrolyzed infant formula eHF1 and eHF1 were the most hydrolyzed with respectively 99% and 98% of their peptides below 1000 Da. The whey-based hydrolyzed formula eHF3 was found to be much less hydrolyzed with only 74% of its peptides below 1000 Da. The residual βLG specific allergenicity of both whey based extensively hydrolyzed infant formulas was similar to the one of the casein based eHF2. The taste of the whey based extensively hydrolyzed infant formula eHF1 was significantly less bitter, less salty, and slightly sweeter than casein based eHF2. Comparison of eHF1 to eHF3 showed that eHF1 was slightly bitter and saltier than eHF3.

## Conclusion

All tested extensively hydrolyzed cow’s milk protein based infant formulas showed very similar low allergenicity *in vitro*, despite significant differences in residual βLG and peptide size profiles. The sensory data underlines that comparable degree of hydrolysis can deliver different sensory basic taste characteristics, important for mother and infant’s acceptance.

## Disclosure of interest

M Rapp: Employee of Nestec, C Martin-Paschoud: Employee of Nestec, S Nutten: Employee of Nestec, C Blanchard: Employee of Nestec, A Panchaud: Employee of Nestec, A Jarvi: Employee of Nestec, M Kuslys: Employee of Nestec.

